# Supplementary material for: Coupling of polymerase-nucleoprotein-RNA in an influenza virus mini ribonucleoprotein complex
Source: Nat Commun. 2025 Nov 4;16:9741. doi: 10.1038/s41467-025-64741-z (PMC12586584; doi:10.1038/s41467-025-64741-z)
Supplement: Supplementary file 1 — Supplementary Information [file 41467_2025_64741_MOESM1_ESM.pdf]

Supplementary Information for:

Coupling of polymerase-nucleoprotein-RNA in influenza virus mini ribonucleoprotein complex

Kang et al.

Contents **Page**

Supplementary Figures 2

Supplementary Figure 1 2

Supplementary Figure 2 4

Supplementary Figure 3 6

Supplementary Figure 4 8

Supplementary Figure 5 10

Supplementary Figure 6 12

Supplementary Figure 7 14

Supplementary Figure 8 14

Supplementary Figure 9 18

Supplementary Figure 10 20

Supplementary Figure 11 22

Supplementary Figure 12 23

Supplementary Table 25

Supplementary Table 1. Cryo-EM data statistics 25

Reference 26

Supplementary Figures

Supplementary Figure 1

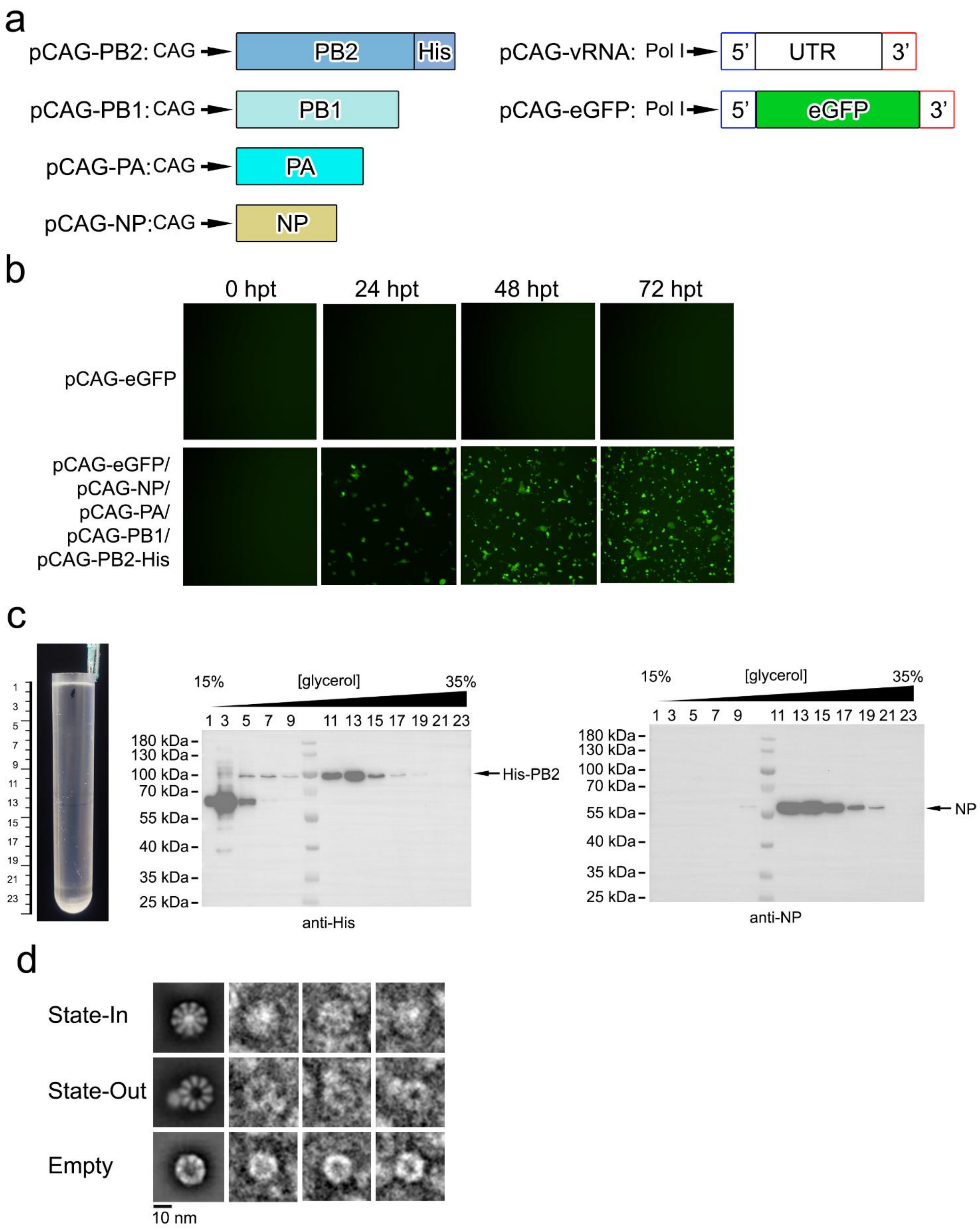

Supplementary Figure 1. Purification of the mini-vRNPs. (a) Schematic diagrams of the plasmids to reconstitute the mini-vRNP in cells. The 5’ and 3’ ends of IAV vRNA are indicated by the blue and red frames. The coding region of PA, PB1, PB2, NP, eGFP or the UTR regions are shown in the same color scheme as in Figure 1. **(b)** COS-1 cells were transfected by the plasmids shown on the left. The expression of eGFP was monitored at 0, 24, 48 and 72 hrs. **(c)** Mini-vRNP was purified using a 15%-35% glycerol gradient centrifugation. The 12 ml of solution in the centrifugation tube was fractionated into 24 samples (0.5 ml for each). The collected samples were analyzed by western-blot using an anti-His-tag or anti-NP antibodies to indicate the existence of PB2 and NP, respectively. **(d)** Three representative classes of three types of particles in 2D classification for the negative-stain EM data are shown in the left panels. The representative raw images of each type of particle are also displayed. **(b, c)** Source data are provided as a Source Data file.

Supplementary Figure 2

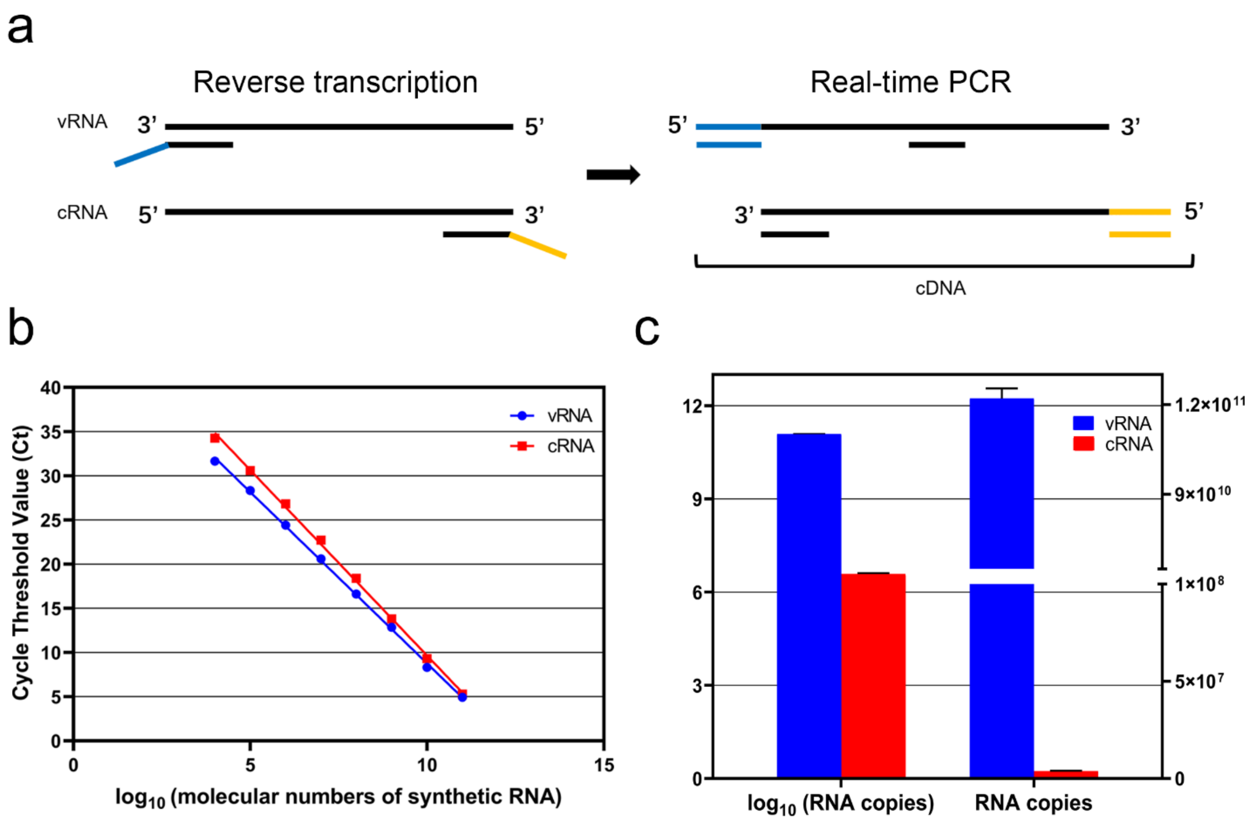

Supplementary Figure 2. Quantification of RNAs in the purified RNPs. (a) Schematic diagram of the real-time PCR with tagged primers to distinguish vRNA and cRNA. **(b)** Standard curve for vRNA (blue) and cRNA (red), generated by plotting the Ct values against the input synthetic RNA molecular numbers. Ten-fold serial dilutions (10^4–10^{11} copies/μl for vRNA and cRNA) of synthetic viral RNA standard were used to generate a standard curve. **(c)** Analysis of vRNA (blue) and cRNA (red) in the purified RNPs using RT-PCR. The average molecular number and standard deviation of triplicate experiments (n=3) are presented as a percentage of the average value of the target type of RNA. Error bars

represent the standard deviation of triplicate experiments. Data are presented as mean values +/- SD. **(b)** Source data are provided as a Source Data file.

Supplementary Figure 3

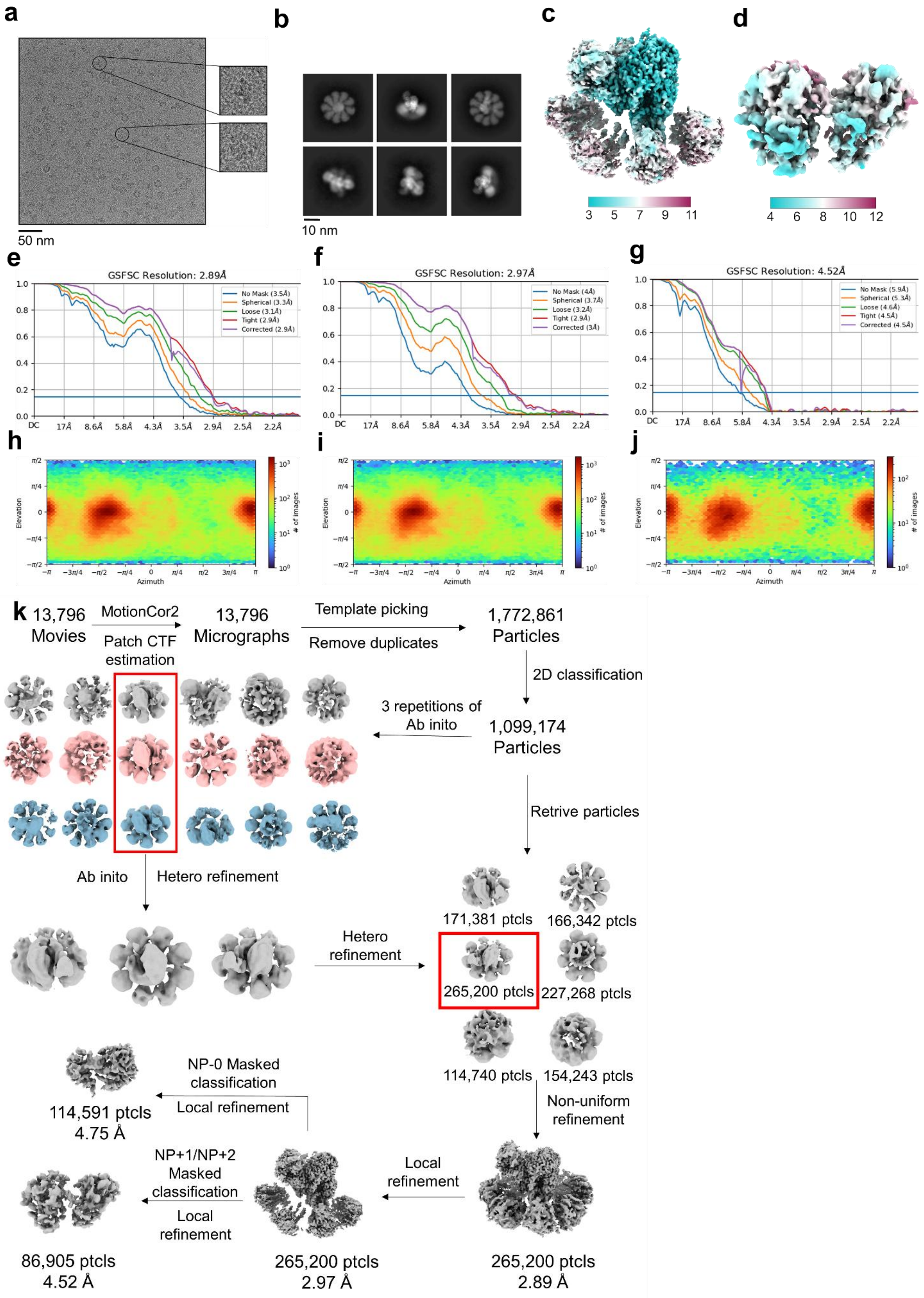

Supplementary Figure 3. Cryo-EM reconstruction of the State-In mini-vRNP. (a) Raw image of RNP particles in vitreous ice recorded at defocus values from -1.0 to -1.8 μm. **(b)** Representative class averages. The edge to each square is ~345 Å. **(c)** Side view of the NP+1:NP+2 local density map colored by local resolution. The local resolution was estimated in cryoSPARC[1]. **d.** Top view of the local-refined density of FluPol:NP-0:NP+1:NP+2:NP-1:RNA unit is colored by local resolution. The local resolution was estimated in cryoSPARC[1]. **(e, f, g)** Fourier shell correlation (FSC) curves of the global maps of FluPol:NP-0:NP+1:NP+2:NP-1:RNA and NP+1:NP+2:RNA units following the gold standard refinement protocol. FSC curves were plotted before and after masking. The blue line represents the 0.143 FSC cutoff. **(h, i, j)** Angular distribution heatmap of particles used for the refinement of the global map, FluPol:NP-0:NP+1:NP+2:NP-1:RNA unit and NP+1:NP+2:RNA unit. **(k)** The map reconstruction pipeline for the State-In mini-vRNP dataset.

Supplementary Figure 4

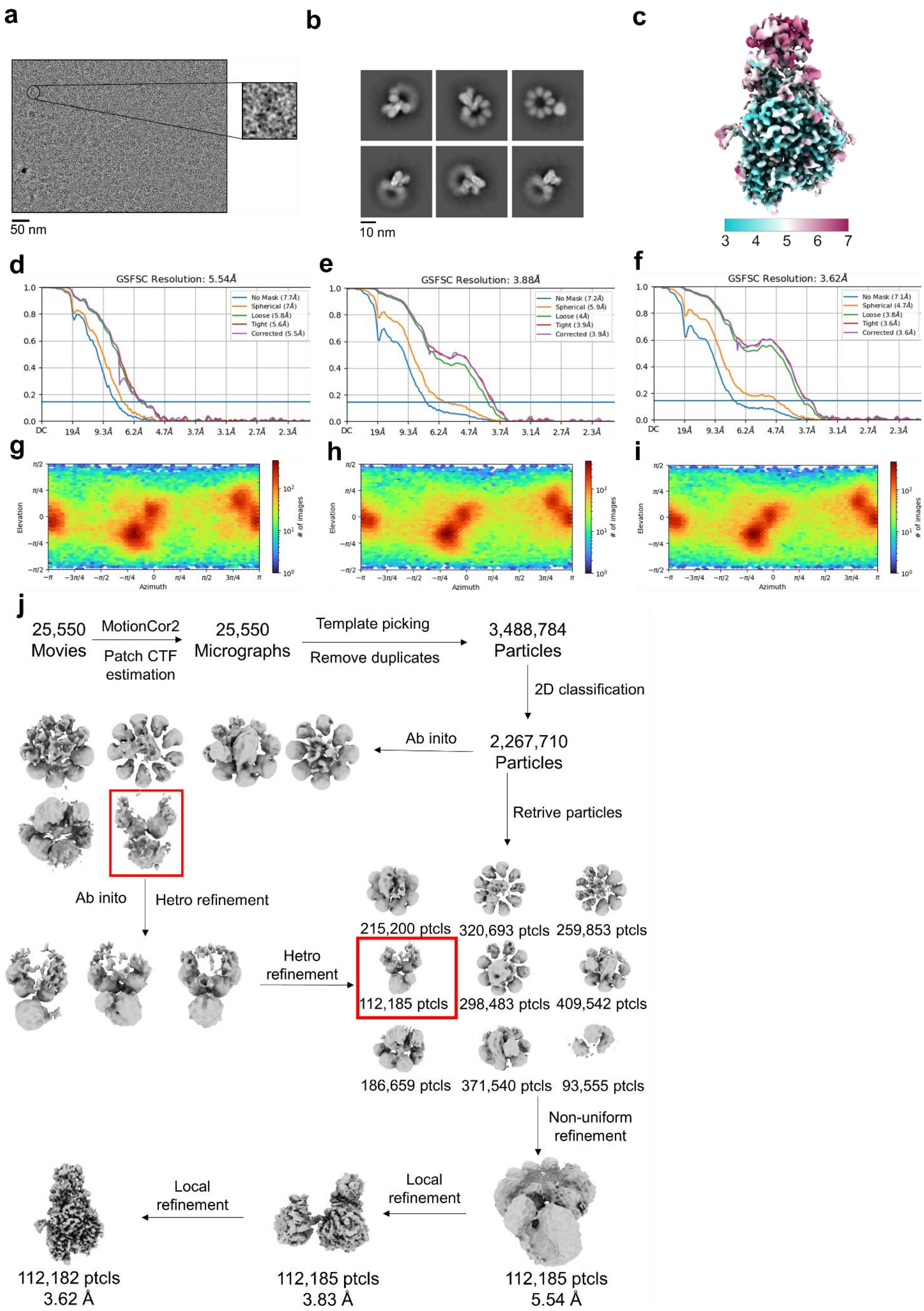

Supplementary Figure 4. Cryo-EM reconstruction of the State-Out mini-vRNP. (a) Raw image of RNP particles in vitreous ice recorded at defocus values from -1.0 to -1.8 µm. **(b)** Representative class averages. The edge of each square is ~466 Å. **(c)** Side view of the local refined density map of FluPol are colored by local resolution. The local resolution was estimated in cryoSPARC[1]. **(d, e, f)** Fourier shell correlation (FSC) curves of the global maps of FluPol:NP-0:RNA unit and FluPol following gold standard refinement. FSC curves were plotted before and after masking. The blue line represents the 0.143 FSC cutoff. **(g, h, i)** Angular distribution heatmap of particles used for the refinement of the global map, FluPol:NP-0:RNA unit and FluPol. **(j)** The map reconstruction pipeline for the State-Out mini-vRNP dataset.

Supplementary Figure 5

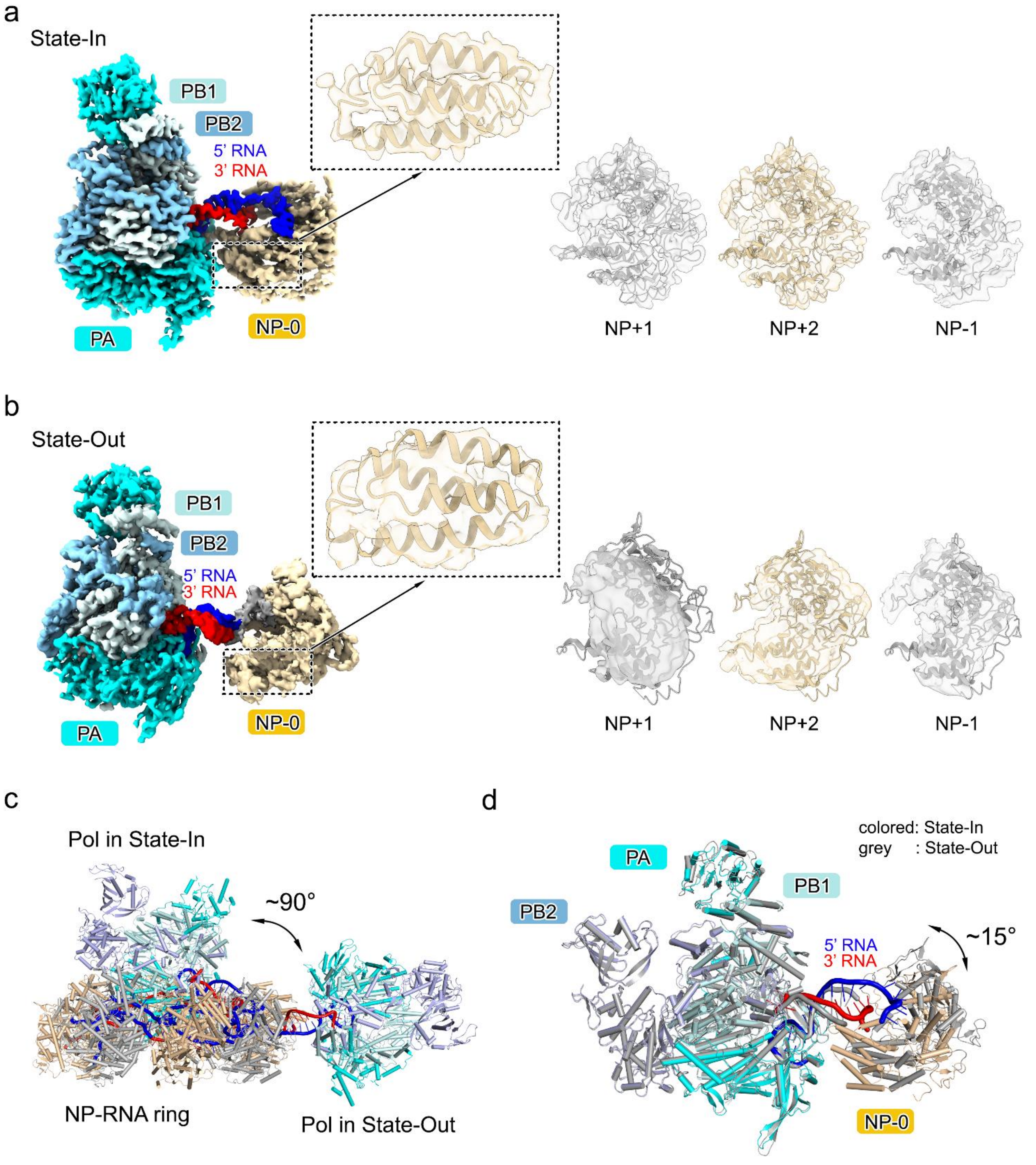

Supplementary Figure 5. Relative orientations of FluPol, NP-0 and RNA in State-In and State-Out. (a, b) The cryo-EM densities covering FluPol:NP-0:RNA, NP+1, NP+2 and NP-1 in State-In (a) or State-Out (b) mini-vRNP. Helical regions in the head domains of NP-0 in

both states are enlarged. The color scheme is the same as in Figure 1. The polypeptides of NP+1, NP+2 and NP-1 are displayed as cartoon diagrams and are covered by cryo-EM densities. It is noteworthy that although the paired 5'/3' termini of vRNA can be traced in the cryo-EM density of State-Out mini-vRNP, the densities of two single strands of RNA bound in the RNA binding groove of NP-0 are clearly visible but do not support building an atomic model. Therefore, the densities corresponding to the RNA in FluPol:NP-0:RNA unit with atomic model are colored in red and blue, while the untraced parts are in grey. **(c)** The models of the mini-vRNP in State-In and State-Out were aligned by using the position of NP-RNA ring as a reference. **(d)** The structures of FluPol:NP-0:RNA of the mini-vRNP in State-In and State-Out are shown as colored and grey cartoons, respectively and are superimposed by using the position of FluPol as a reference.

Supplementary Figure 6

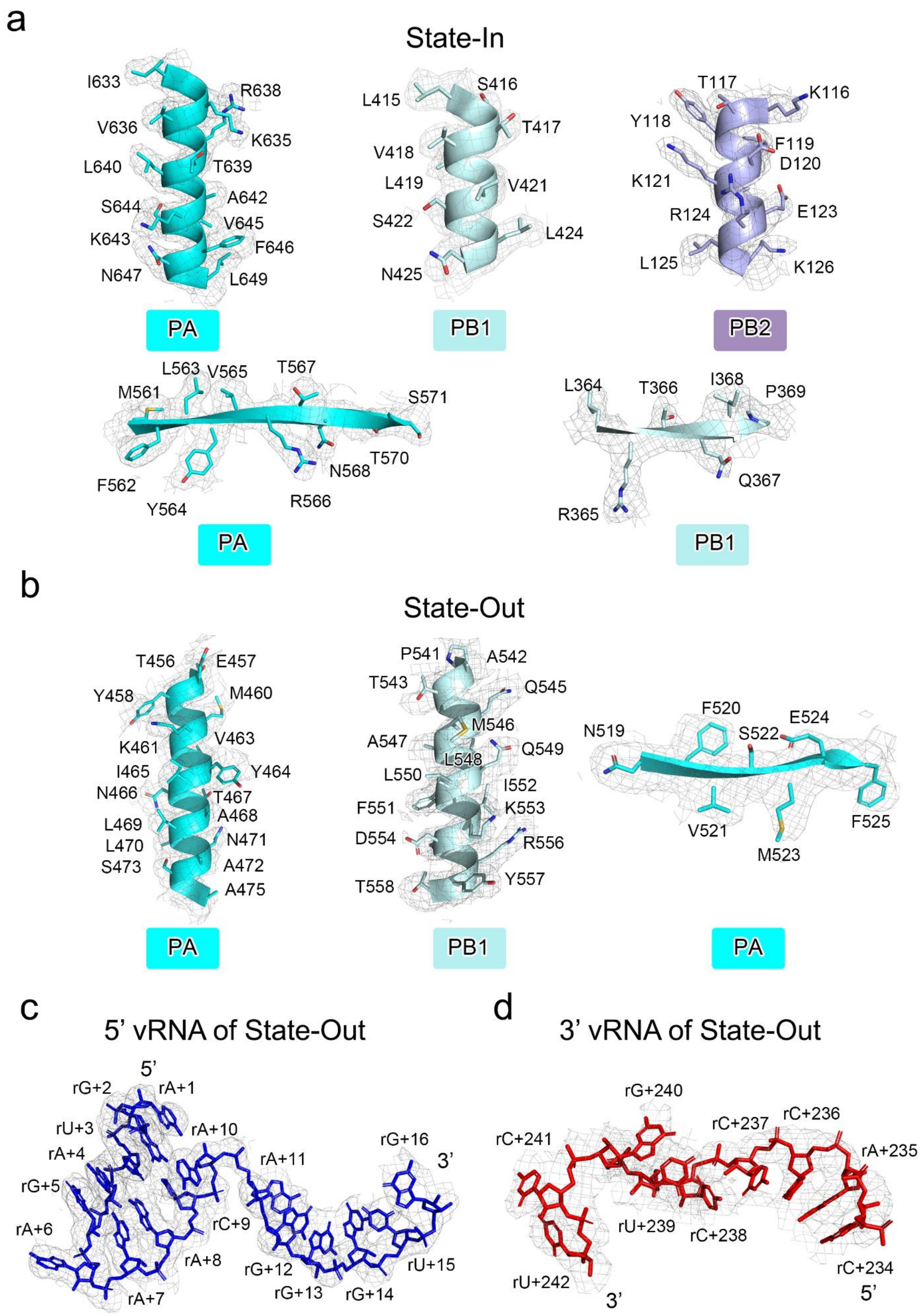

Supplementary Figure 6. Representative densities of State-In and State-Out mini-vRNP reconstructions (contoured at 5σ). (a, b) The cryo-EM densities for representative parts of FluPol in State-In (a) and in State-Out (b) are shown in grey mesh. The main chains are shown in cartoon representation and the side chains are shown as sticks. PA is in cyan, PB1 is in pale cyan and PB2 is in light blue. **(c, d)** The cryo-EM densities for vRNA 5' nucleotides (c) and vRNA 3' nucleotides (d) in State-Out structures are shown in grey mesh. The nucleotides are shown as sticks, 5' RNA is in blue and the 3' RNA is in red.

Supplementary Figure 7

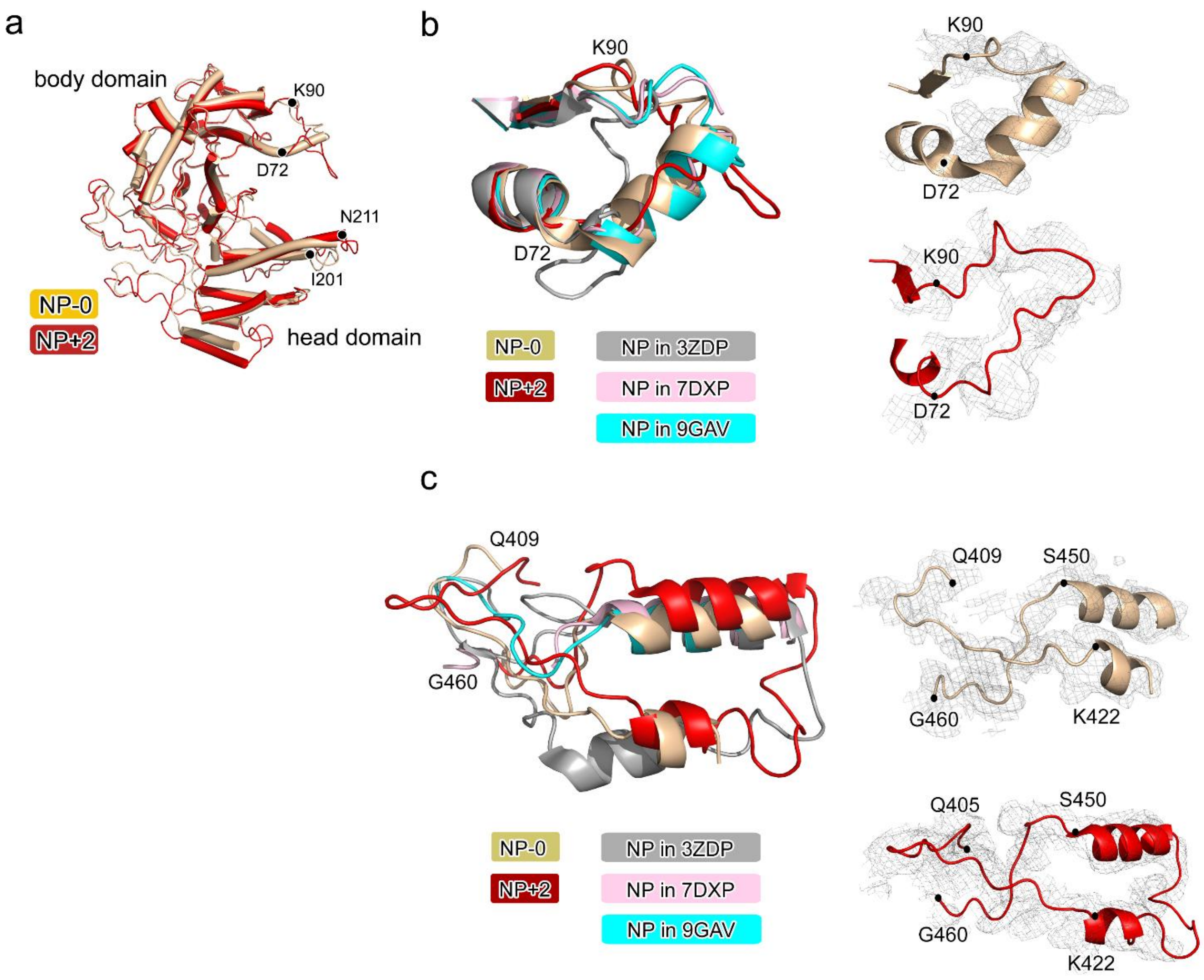

Supplementary Figure 7. Structure comparison of NP-0 and NP+2 with the monomeric NP[2] (PDB code: 3ZDP), the NP which bound to 3-mer RNA[3] (PDB code: 7DXP) and the NP which bound to 18-mer RNA[4] (PDB code: 9GAV). (a) Structure comparison of NP-0 and NP+2. The polypeptides of NP-0 (in FluPol:NP-0:RNA unit) and NP+2 are shown as wheat and red cartoons. The starting and ending residues for two loop regions with a conformational change in NPs are labeled. **(b, c)** Conformational changes occur in two regions of NP-0 and NP+2. The first region contains the D70-K90 loop (b) and the second region contains polypeptides Q405-G460 which includes the tail loop (c). The polypeptides of NP-0 (in FluPol:NP-0:RNA unit), NP+2, NP in 3ZDP, NP in 7DXP and NP in 9GAV are shown in wheat, red, grey, light pink and cyan cartoons, respectively. The cryo-EM densities

for NP-0 and NP+2 in the two regions are shown in grey mesh. The starting and ending residues for two regions with a conformational change in NPs are labeled.

Supplementary Figure 8

a

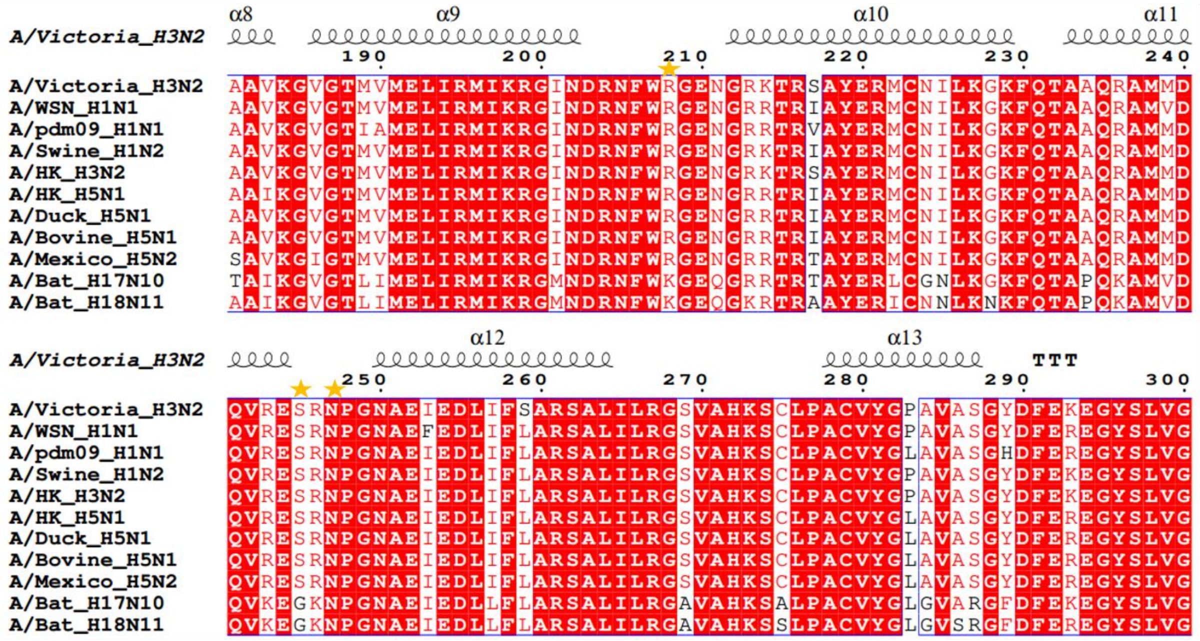

b

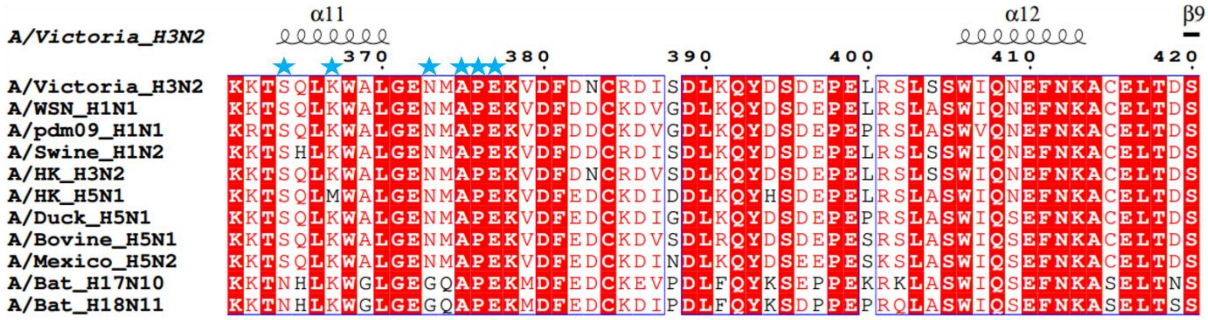

Supplementary Figure 8. Sequence alignment of NP (a) / PA (b) from representative influenza A viruses. A/Victoria_H3N2, A/Victoria/3/1975(H3N2) (AFG98999.1 and AFG99002.1); A/WSN_H1N1, A/Wilson-Smith/1933(H1N1) (ABD77800.1 and ABD77803.1); A/pdm09_H1N1, A/Lyon/969/2009(H1N1) (AGI63956.1 and AGI63954.1); A/Swine_H1N2, A/NWS-F(NWS/1934-Rockefeller Institute/5/1957)(H1N2) (ACV49659.1 and ACV49662.1); A/HK_H3N2, A/Hong Kong/01/1968(H3N2) (APO40570.1 and APO40567.1); A/HK_H5N1, A/Hong Kong/156/97(H5N1) (CAC19704.1 and CAB95840.1); A/Duck_H5N1, A/mallard/California/2535P/2011(H5N1) (AGE08771.1 and AGE08774.1); A/ Bovine_H5N1, A/dairy_cow/Kansas/SM-3/2024(H5N1)(XDF03184.1 and XDF03180.1); A/Mexico_H5N2, A/State_of_Mexico/2024(H5N2) (XBP46197.1 and XBU81422.1);

A/Bat_H17N10, A/little yellow-shouldered bat/Guatemala /060/2010(H17N10) (AFC35439.1 and AFC35437.1); A/Bat_H18N11, A/flat-faced bat/Peru/033/2010(H18N11) (AGX84935.1 and AGX84933.1). The orange and blue stars indicate the position of the residues involved in the NP-PA interactions, respectively. The sequences were aligned using Clustal W[5] and manually adjusted, the figure was drawn with ESPript[6].

Supplementary Figure 9

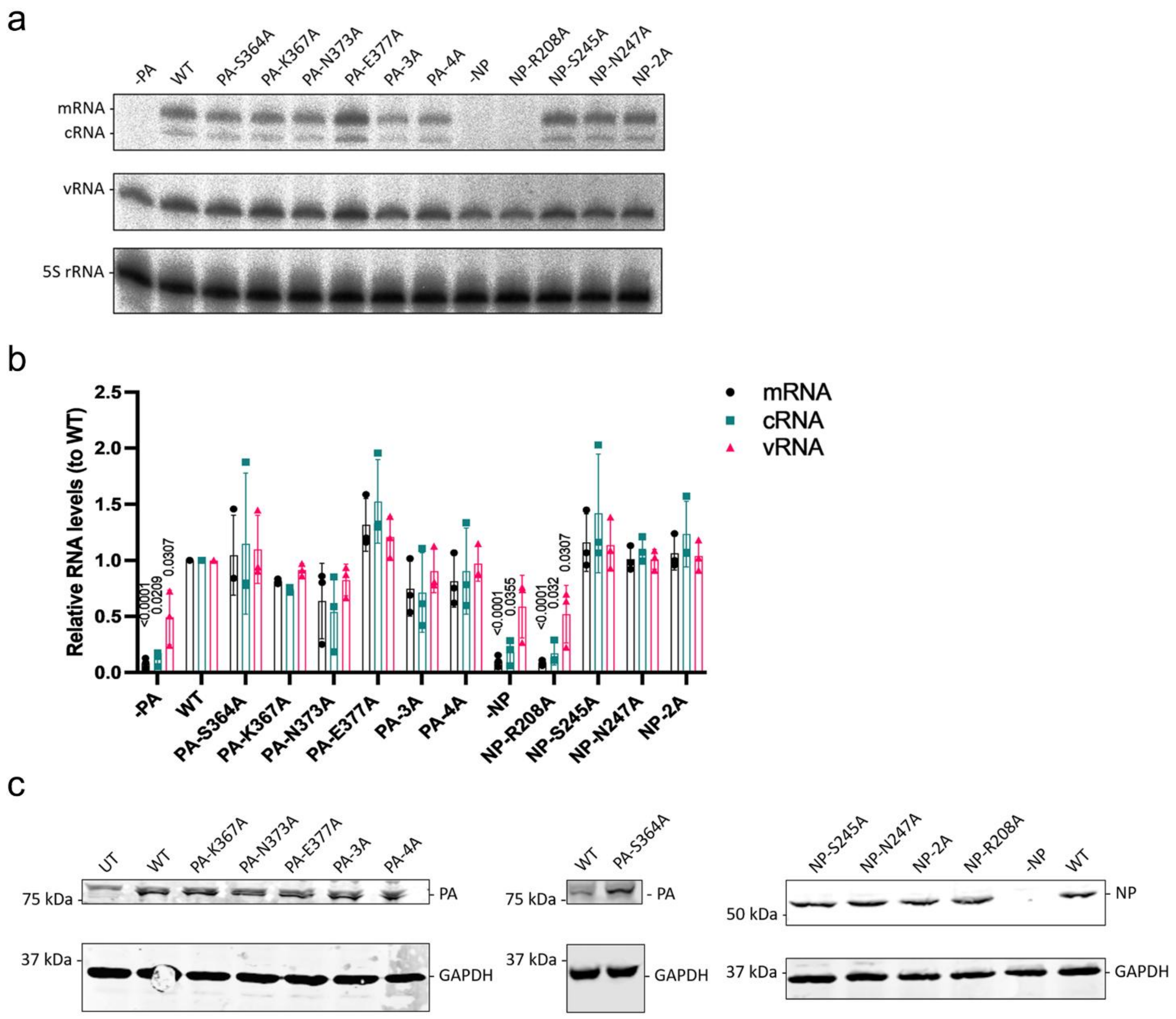

Supplementary Figure 9. Effect of PA and NP mutations on influenza A/WSN/33 (H1N1) vRNP activity. (a) A neuraminidase (NA) vRNA template was used. Total RNA was isolated at 12 h post-transfection and analyzed by primer extension. **(b)** Quantification of viral RNAs by primer extension. Error bars represent standard deviation from three independent experiments (n=3). Data are presented as mean values +/- SD. **(c)** Western blot analysis of the expression of mutant proteins in vRNP reconstitution assays. WT, wild

type; PA-3A, PA-S364A/K367A/N373A; PA-4A, PA-S364A/K367A/N373A/E377A; NP-2A, NP-S245A/N247A. **(a, b, c)** Source data are provided as a Source Data file.

Supplementary Figure 10

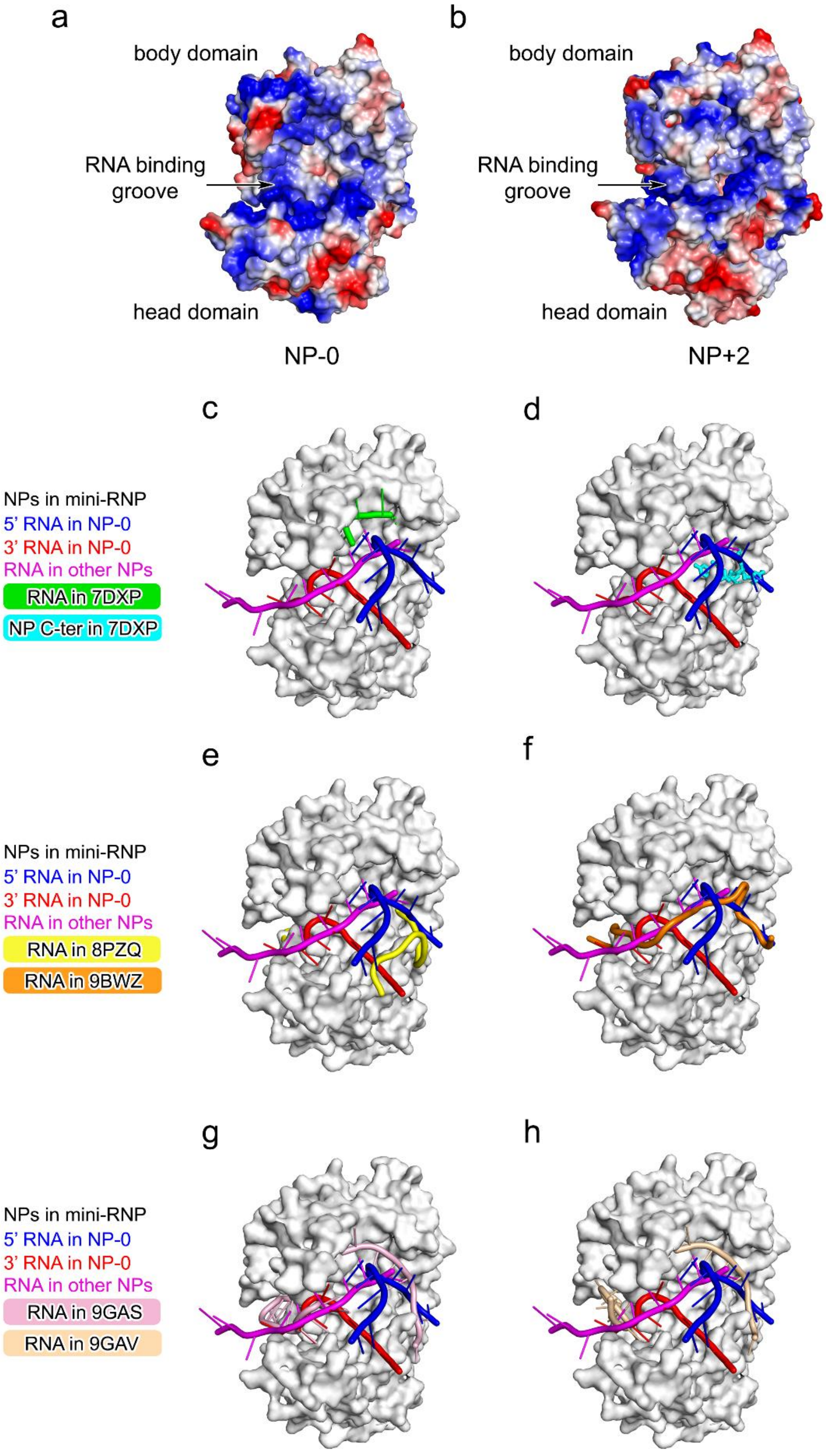

Supplementary Figure 10. Comparison of NP-RNA complexes. (a, b) NP-0 (a) and NP+2 (b) shown as electrostatic surfaces and shown in the same orientation. The RNA binding grooves are labeled and indicated by arrows. **(c, d)** A previously reported crystal structure of NP:RNA complex[3] (PDB: 7DXP) is aligned with NP-0:RNA and NP+2:RNA in State-In mini-vRNP. The polypeptide of NP is represented by a white molecular surface. The 5' and 3' RNA in State-In mini-vRNP are shown as blue and red cartoons, respectively in (c) and (d), while the RNA in crystal structure of NP:RNA complex (PDB: 7DXP) is shown as a green cartoon in (c). The last six C-terminal residues of NP in the crystal structure of NP:RNA complex (PDB: 7DXP) are displayed as cyan sticks in (d). **(e-h)** Four reported cryo-EM structures of NP:RNA complex[4,7,8] aligned with NP-0:RNA and NP+2:RNA in State-In mini-vRNP. The polypeptide of NP is represented by a white molecular surface. The 5' and 3' RNA in State-In mini-vRNP are shown as blue and red cartoons, respectively in (e-h), the linear RNA in NP+2:RNA is show as magenta cartoon. The RNA in 8PZQ (e), 9BWZ (f), 9GAS (g) and 9GAV (h) are shown as yellow, orange, lightpink and wheat cartoons, respectively.

Supplementary Figure 11

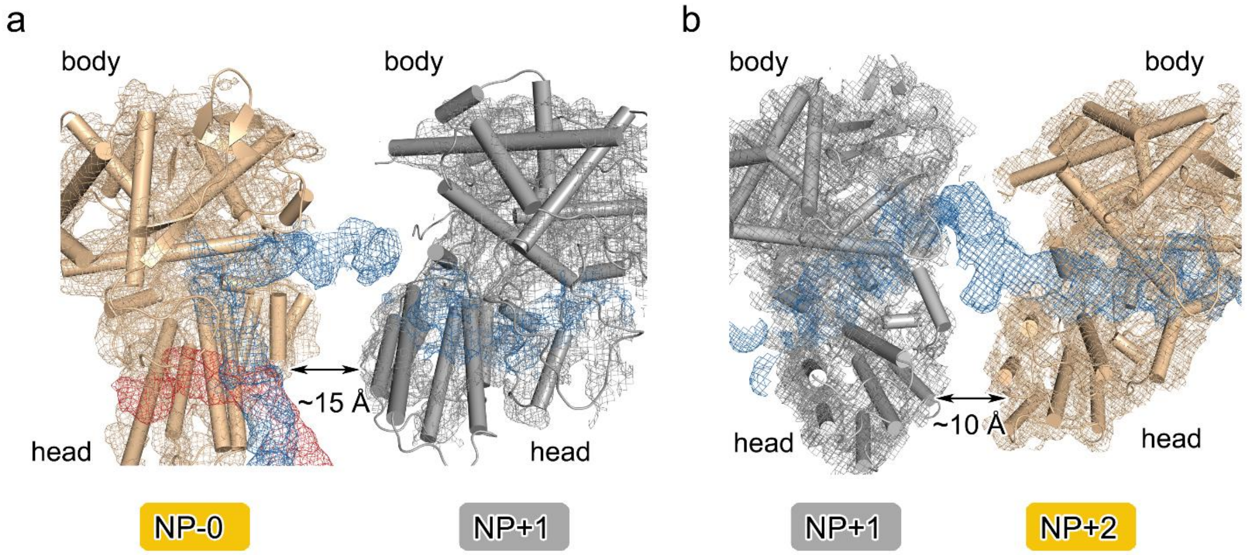

Supplementary Figure 11. Distances between NPs in the mini-vRNP. The polypeptides of NP-0/NP+1 (a) and NP+1/NP+2 (b) are covered by cryo-EM densities. The cryo-EM densities for 3' terminus of vRNA, 5' terminus of vRNA in FluPol:NP-0:RNA unit are shown as red and blue mesh, respectively while the density for vRNA in the 5' direction in NP+1:NP+2:RNA unit is shown as blue mesh. The approximately closest distance for the NPs are indicated with labels.

Supplementary Figure 12

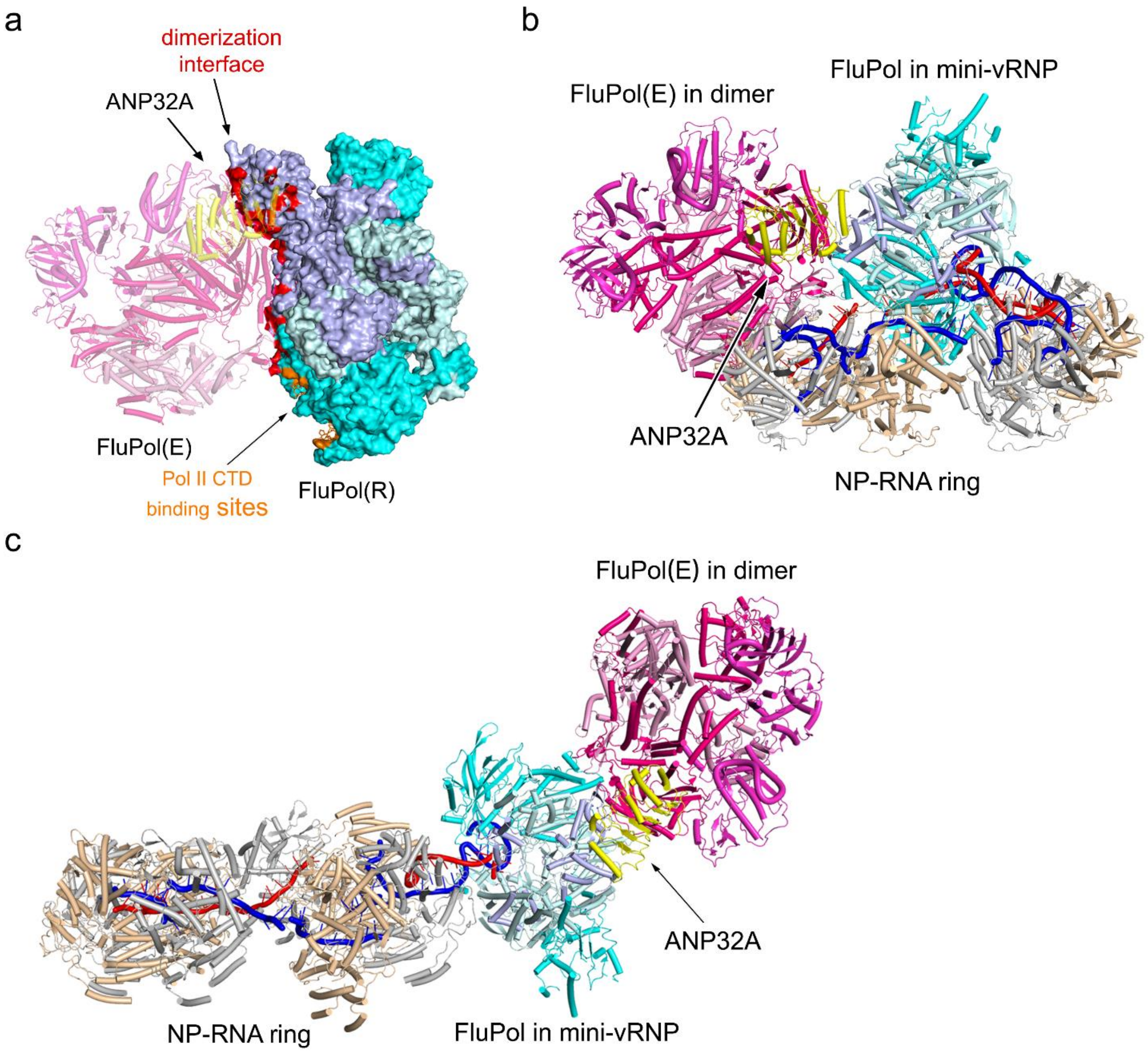

Supplementary Figure 12. Dimerization and Pol II-interacting interfaces on FluPol. (a) Interfaces for FluPol dimerization and FluPol interaction with Pol II CTD. In a previously reported structure of the FluPol dimer with ANP32A[9] (PDB code: 8RMR), the replicating FluPol (FluPol(R)) is shown as a colored molecular surface, while the encapsidating FluPol (FluPol(E)) and ANP32A are shown as a semi-transparent cartoon. The dimerization interface of FluPol is highlighted by the red surface of FluPol(R). The interface for FluPol interacting with Pol II CTD retrieved from a previously reported structure [10] (PDB code: 5M3H) is highlighted in orange and the residues of Pol II CTD are shown as orange sticks.

(b, c) The FluPol dimer aligned to the mini-vRNP in State-In (b) or State-Out (c) structures using FluPol in the mini-vRNP as a reference.

Supplementary Table

Supplementary Table 1. Cryo-EM data statistics

	State-In mini-vRNP			State-Out mini-vRNP
**Data collection and processing**				
Magnification	130,000			22,500
Voltage (kV)	300			300
Electron exposure (e–/Å^2)	60			60
Defocus range (μm)	-2.0 to -1.0			-2.0 to -1.0
Pixel size (Å)	0.96			1.06
Symmetry imposed	C1			C1
Initial particle images (no.)	1,772,861			2,267,710
Final particle images (no.)	265,200			112,185
Map resolution (Å)<br>FSC threshold	2.89<br>0.143			5.54<br>0.143
**Refinement**				
	FluPol:NP-0:RNA	NP+1:NP+2: RNA	NP-0:RNA	FluPol
Initial model used (PDB)	5M3H<br>3ZDP	3ZDP	7NT8	5M3H
Model resolution (Å)<br>FSC threshold	2.97<br>0.143	4.52<br>0.143	4.75<br>0.143	3.62<br>0.143
Map sharpening *B* factor (Å^2)	-84.5	-202.7	-262.3	-95.7
Model composition<br>Non-hydrogen atoms<br>Protein residues<br>Nucleotide	 17837<br>2122<br>38	 3792<br>453<br>10	 4154<br>420<br>40	 14007<br>1671<br>25
B factors (Å^2)<br>Protein<br>Nucleotide	 60.50<br>53.46	 109.9<br>51.72	 13.21<br>83.72	 73.69<br>75.52
R.m.s. deviations<br>Bond lengths (Å)<br>Bond angles (°)	 0.007<br>1.156	 0.009<br>1.300	 0.006<br>1.035	 0.007<br>1.002
Validation<br>MolProbity score<br>Clashscore<br>Rotamers outliers (%)	 3.04<br>22.85<br>7.84	 3.60<br>46.56<br>14.10	 3.07<br>33.19<br>4.26	 2.85<br>23.88<br>3.42
Ramachandran plot<br>Favored (%)<br>Allowed (%)<br>Outliers (%)	 93.4<br>6.3<br>0.3	 89.6<br>10.2<br>0.2	 92.0<br>7.7<br>0.2	 91.7<br>8.3<br>0

Reference

1 Punjani, A., Rubinstein, J. L., Fleet, D. J. & Brubaker, M. A. cryoSPARC: algorithms for rapid unsupervised cryo-EM structure determination. *Nat Methods* **14**, 290-296 (2017). https://doi.org/10.1038/nmeth.4169

2 Chenavas, S. *et al.* Monomeric nucleoprotein of influenza A virus. *PLoS pathogens* **9**, e1003275 (2013). https://doi.org/10.1371/journal.ppat.1003275

3 Tang, Y. S., Xu, S., Chen, Y. W., Wang, J. H. & Shaw, P. C. Crystal structures of influenza nucleoprotein complexed with nucleic acid provide insights into the mechanism of RNA interaction. *Nucleic acids research* **49**, 4144-4154 (2021). https://doi.org/10.1093/nar/gkab203

4 Chenavier, F. *et al.* Influenza a virus antiparallel helical nucleocapsid-like pseudo-atomic structure. *Nucleic acids research* **53** (2025). https://doi.org/10.1093/nar/gkae1211

5 Larkin, M. A. *et al.* Clustal W and Clustal X version 2.0. *Bioinformatics* **23**, 2947-2948 (2007). https://doi.org/10.1093/bioinformatics/btm404

6 Gouet, P., Courcelle, E., Stuart, D. I. & Métoz, F. ESPript: analysis of multiple sequence alignments in PostScript. *Bioinformatics* **15**, 305-308 (1999). https://doi.org/10.1093/bioinformatics/15.4.305

7 Chenavier, F. *et al.* Cryo-EM structure of influenza helical nucleocapsid reveals NP-NP and NP-RNA interactions as a model for the genome encapsidation. *Sci Adv* **9**, eadj9974 (2023). https://doi.org/10.1126/sciadv.adj9974

8 Peng, R. *et al.* Molecular basis of influenza ribonucleoprotein complex assembly and processive RNA synthesis. *Science (New York, N.Y.)* **388**, eadq7597 (2025). https://doi.org/10.1126/science.adq7597

9 Arragain, B. *et al.* Structures of influenza A and B replication complexes give insight into avian to human host adaptation and reveal a role of ANP32 as an electrostatic chaperone for the apo-polymerase. *Nature communications* **15**, 6910 (2024). https://doi.org/10.1038/s41467-024-51007-3

10 Lukarska, M. *et al.* Structural basis of an essential interaction between influenza polymerase and Pol II CTD. *Nature* **541**, 117-121 (2017). https://doi.org/10.1038/nature20594
