# Supplementary material for: Coupling of polymerase-nucleoprotein-RNA in an influenza virus mini ribonucleoprotein complex
Source: Nat Commun. 2025 Nov 4;16:9741. doi: 10.1038/s41467-025-64741-z (PMC12586584; doi:10.1038/s41467-025-64741-z)
Supplement: Supplementary file 2 — Description of Additional Supplementary Files [file 41467_2025_64741_MOESM2_ESM.pdf]

<u>Description of Additional Supplementary Files</u>

File name: Supplementary Movie 1
Description: Cryo-EM densities of the mini-vRNP in the State-In conformation.

File name: Supplementary Movie 2
Description: Structural model of the mini-vRNP in the State-In conformation.

File name: Supplementary Movie 3
Description: Cryo-EM densities of the mini-vRNP in the State-Out conformation.

File name: Supplementary Movie 4
Description: Structural model of the mini-vRNP in the State-Out conformation.

File name: Supplementary Movie 5
Description: Cryo-EM densities of the FluPol:NP-0:RNA unit within the State-In conformation of mini-vRNP.

File name: Supplementary Movie 6
Description: Cryo-EM densities of the FluPol:NP-0:RNA unit within the State-Out conformation of mini-vRNP.
