# Supplementary material for: Coupling of polymerase-nucleoprotein-RNA in an influenza virus mini ribonucleoprotein complex
Source: Nat Commun. 2025 Nov 4;16:9741. doi: 10.1038/s41467-025-64741-z (PMC12586584; doi:10.1038/s41467-025-64741-z)
Supplement: Supplementary file 9 — Reporting Summary [file 41467_2025_64741_MOESM9_ESM.pdf]

nature portfolio

Corresponding author(s): Ervin Fodor, Zihe Rao, Zhiyong Lou

Last updated by author(s): Aug 15, 2025

Reporting Summary

Nature Portfolio wishes to improve the reproducibility of the work that we publish. This form provides structure for consistency and transparency in reporting. For further information on Nature Portfolio policies, see our Editorial Policies and the Editorial Policy Checklist.

Statistics

For all statistical analyses, confirm that the following items are present in the figure legend, table legend, main text, or Methods section.

n/a	Confirmed	
☐	☒	The exact sample size (*n*) for each experimental group/condition, given as a discrete number and unit of measurement
☐	☒	A statement on whether measurements were taken from distinct samples or whether the same sample was measured repeatedly
☐	☒	The statistical test(s) used AND whether they are one- or two-sided<br>*Only common tests should be described solely by name; describe more complex techniques in the Methods section.*
☒	☐	A description of all covariates tested
☒	☐	A description of any assumptions or corrections, such as tests of normality and adjustment for multiple comparisons
☐	☒	A full description of the statistical parameters including central tendency (e.g. means) or other basic estimates (e.g. regression coefficient) AND variation (e.g. standard deviation) or associated estimates of uncertainty (e.g. confidence intervals)
☐	☒	For null hypothesis testing, the test statistic (e.g. *F*, *t*, *r*) with confidence intervals, effect sizes, degrees of freedom and *P* value noted<br>*Give P values as exact values whenever suitable.*
☒	☐	For Bayesian analysis, information on the choice of priors and Markov chain Monte Carlo settings
☒	☐	For hierarchical and complex designs, identification of the appropriate level for tests and full reporting of outcomes
☒	☐	Estimates of effect sizes (e.g. Cohen's *d*, Pearson's *r*), indicating how they were calculated

Our web collection on statistics for biologists contains articles on many of the points above.

Software and code

Policy information about availability of computer code

Data collection	SerialEM 4.0
Data analysis	cryoSPARC v3.2.0, relion 3.1.2Phenix 1.19.2, Coot 0.9.8, ChimeraX 1.6, GraphPad Prism 10, GelAnalyzer V 19.1, deepEMhancer v1.0

For manuscripts utilizing custom algorithms or software that are central to the research but not yet described in published literature, software must be made available to editors and reviewers. We strongly encourage code deposition in a community repository (e.g. GitHub). See the Nature Portfolio guidelines for submitting code & software for further information.

Data

Policy information about availability of data

All manuscripts must include a data availability statement. This statement should provide the following information, where applicable:

- Accession codes, unique identifiers, or web links for publicly available datasets
- A description of any restrictions on data availability
- For clinical datasets or third party data, please ensure that the statement adheres to our policy

The cryo-EM density maps and the structures were deposited into the Electron Microscopy Data Bank (EMDB) and Protein Data Bank (PDB) with the accession numbers EMD-64766 and 9V44 for FluPol_NP-0_RNA of State In, EMD-64770 and 9V48 for NP-0_RNA of State In, EMD-64769 and 99V47 for NP+1_NP+2_RNA of State In, EMD-64768 and 9V46 for FluPol of State Out. The previously published influenza structures used in this study are available in the Protein Data Bank under

accession codes 7DXP, 5M3H, 3ZDP and 7NT8. Source data are provided with this paper.

Research involving human participants, their data, or biological material

Policy information about studies with human participants or human data. See also policy information about sex, gender (identity/presentation), and sexual orientation and race, ethnicity and racism.

Reporting on sex and gender	*Use the terms sex (biological attribute) and gender (shaped by social and cultural circumstances) carefully in order to avoid confusing both terms. Indicate if findings apply to only one sex or gender; describe whether sex and gender were considered in study design; whether sex and/or gender was determined based on self-reporting or assigned and methods used. Provide in the source data disaggregated sex and gender data, where this information has been collected, and if consent has been obtained for sharing of individual-level data; provide overall numbers in this Reporting Summary. Please state if this information has not been collected. Report sex- and gender-based analyses where performed, justify reasons for lack of sex- and gender-based analysis.*
Reporting on race, ethnicity, or other socially relevant groupings	*Please specify the socially constructed or socially relevant categorization variable(s) used in your manuscript and explain why they were used. Please note that such variables should not be used as proxies for other socially constructed/relevant variables (for example, race or ethnicity should not be used as a proxy for socioeconomic status). Provide clear definitions of the relevant terms used, how they were provided (by the participants/respondents, the researchers, or third parties), and the method(s) used to classify people into the different categories (e.g. self-report, census or administrative data, social media data, etc.) Please provide details about how you controlled for confounding variables in your analyses.*
Population characteristics	*Describe the covariate-relevant population characteristics of the human research participants (e.g. age, genotypic information, past and current diagnosis and treatment categories). If you filled out the behavioural & social sciences study design questions and have nothing to add here, write "See above."*
Recruitment	*Describe how participants were recruited. Outline any potential self-selection bias or other biases that may be present and how these are likely to impact results.*
Ethics oversight	*Identify the organization(s) that approved the study protocol.*

Note that full information on the approval of the study protocol must also be provided in the manuscript.

Field-specific reporting

Please select the one below that is the best fit for your research. If you are not sure, read the appropriate sections before making your selection.

☒ Life sciences ☐ Behavioural & social sciences ☐ Ecological, evolutionary & environmental sciences

For a reference copy of the document with all sections, see nature.com/documents/nr-reporting-summary-flat.pdf

Life sciences study design

All studies must disclose on these points even when the disclosure is negative.

Sample size	Protein sample size were decided as needed by cryo-EM sample preparation.
Data exclusions	During cryo-EM data processing, some cryo-EM movies were discarded owing to poor ice quality, excessive movement or defocus. Particles with poor quality were removed during 2D/3D classification. Otherwise no data were excluded.
Replication	In vitro functional assays and real time PCR were replicated at least twice.
Randomization	Not relevent for protein structure determination or in vitro assay. The protein purification, cell culture, the preparation of cryo-EM sample and data collection were performed following the standard procedure.
Blinding	The structure analysis using cryo-EM and functional analysis involving biochemical studies. Consequently, blinding is not relevant to this study.

Reporting for specific materials, systems and methods

We require information from authors about some types of materials, experimental systems and methods used in many studies. Here, indicate whether each material, system or method listed is relevant to your study. If you are not sure if a list item applies to your research, read the appropriate section before selecting a response.

Materials & experimental systems

n/a	Involved in the study	
☐	☒	Antibodies
☐	☒	Eukaryotic cell lines
☒	☐	Palaeontology and archaeology
☒	☐	Animals and other organisms
☒	☐	Clinical data
☒	☐	Dual use research of concern
☒	☐	Plants

Methods

n/a	Involved in the study	
☒	☐	ChIP-seq
☒	☐	Flow cytometry
☒	☐	MRI-based neuroimaging

Antibodies

Antibodies used

anti-NP , GeneTex, GTX125989
anti-PA, gift from T. Toyoda, Kurume University, Kurume, Japan
anti-GAPDH, Cell Signalling Technology ,14C10
conjugated IRDye 800CW goat anti-rabbit IgG, Li-COR, 926-32211
conjugated IRDye 680LT anti-mouse IgG, Li-COR, 926-32220
Anti-6x His Tag antibody, Sangon Biotech, D191001
HRP-conjugated Goat Anti-Mouse IgG, Sangon Biotech, D110087
Influenza A Nucleoprotein / NP Antibody, Mouse MAb SinoBiological, 11675-MM03T

Validation

anti-PA is a gift from T. Toyoda and it was validated in several publications. (PMID: 15827195, 15308710, 10881677 etc.)
other antibodies validated by the company:
anti-NP(https://www.genetex.com/Product/Detail/Influenza-A-virus-Nucleoprotein-antibody/GTX125989)
anti-GAPDH(https://www.cellsignal.com/products/primary-antibodies/gapdh-14c10-rabbit-mab/2118)
Anti-6x His Tag antibody(https://store.sangon.com/productDetail?productInfo.code=D191001)
Influenza A Nucleoprotein / NP Antibody (https://www.sinobiological.com/antibodies/nucleocapsid-np-11675-mm03t)

Eukaryotic cell lines

Policy information about cell lines and Sex and Gender in Research

Cell line source(s)

HEK293F cells were obtained from Thermo Fisher Scientific (Cat# R79007)
COS-1 cells were obtained from ATCC(Cat# CRL-1650)
HEK 293T cells were sourced from the Cell Bank of the Sir William Dunn School of Pathology, University of Oxford

Authentication

All cell lines were frequently checked for cellular morphologies, growth rates and function

Mycoplasma contamination

All cell lines tested negative for mycoplamsa contamination.

Commonly misidentified lines
(See ICLAC register)

Name any commonly misidentified cell lines used in the study and provide a rationale for their use.

Plants

Seed stocks

Report on the source of all seed stocks or other plant material used. If applicable, state the seed stock centre and catalogue number. If plant specimens were collected from the field, describe the collection location, date and sampling procedures.

Novel plant genotypes

Describe the methods by which all novel plant genotypes were produced. This includes those generated by transgenic approaches, gene editing, chemical/radiation-based mutagenesis and hybridization. For transgenic lines, describe the transformation method, the number of independent lines analyzed and the generation upon which experiments were performed. For gene-edited lines, describe the editor used, the endogenous sequence targeted for editing, the targeting guide RNA sequence (if applicable) and how the editor was applied.

Authentication

Describe any authentication procedures for each seed stock used or novel genotype generated. Describe any experiments used to assess the effect of a mutation and, where applicable, how potential secondary effects (e.g. second site T-DNA insertions, mosiacism, off-target gene editing) were examined.
